# Differentiation of Mouse Embryonic Stem Cells into Endoderm without Embryoid Body Formation

**DOI:** 10.1371/journal.pone.0014146

**Published:** 2010-11-30

**Authors:** Peter T. W. Kim, Brad G. Hoffman, Annette Plesner, Cheryl D. Helgason, C. Bruce Verchere, Stephen W. Chung, Garth L. Warnock, Alice L. F. Mui, Christopher J. Ong

**Affiliations:** 1 Department of Surgery, University of British Columbia, Vancouver, Canada; 2 Department of Pathology and Laboratory Medicine, University of British Columbia, Vancouver, Canada; University of Bremen, Germany

## Abstract

Pluripotent embryonic stem cells hold a great promise as an unlimited source of tissue for treatment of chronic diseases such as Type 1 diabetes. Herein, we describe a protocol using all-*trans*-retinoic acid, basic fibroblast growth factor and dibutyryl cAMP (DBcAMP) in the absence of embryoid body formation, for differentiation of murine embryonic stem cells into definitive endoderm that may serve as pancreatic precursors. The produced cells were analyzed by quantitative PCR, immunohistochemistry and static insulin release assay for markers of trilaminar embryo, and pancreas. Differentiated cells displayed increased Sox17 and Foxa2 expression consistent with definitive endoderm production. There was minimal production of Sox7, an extraembryonic endoderm marker, and Oct4, a marker of pluripotency. There was minimal mesoderm or neuroectoderm formation based on expression levels of the markers brachyury and Sox1, respectively. Various assays revealed that the cell clusters generated by this protocol express markers of the pancreatic lineage including insulin I, insulin II, C-peptide, PDX-1, carboxypeptidase E, pan-cytokeratin, amylase, glucagon, PAX6, Ngn3 and Nkx6.1. This protocol using all-*trans*-retinoic acid, DBcAMP, in the absence of embryoid bodies, generated cells that have features of definitive endoderm that may serve as pancreatic endocrine precursors.

## Introduction

Embryonic stem (ES) cells are pluripotent cells with the ability to differentiate *in vivo* and *in vitro* into all cell types of the embryo proper. These cells represent a potentially unlimited source of differentiated cells or tissue for transplantation for common diseases such as type 1 diabetes mellitus. During embryogenesis *in vivo*, the liver and the pancreas arise from the definitive endoderm [1] which is produced through the process of gastrulation from the embryonic ectoderm of the epiblast [2]. Efficient production of definitive endoderm would allow for on-demand production of pancreatic tissues.

Efforts have been devoted to producing glucose responsive insulin producing (GRIP) cells from ES cells. Although initial studies on the generation of GRIP cells seemed encouraging [3], [4], later work revealed that observed insulin expression resulted from insulin uptake from the media rather than *de novo* synthesis [5], [6]. More recently, D'amour et al., by using activin A, exendin, cyclopamine and retinoic acid, showed that human embryonic stem cells can differentiate into endocrine cells capable of synthesizing pancreatic hormones [7]. The same group of investigators went on to produce cells that express insulin and C-peptide [8]. Since then, other scientists have differentiated human ES cells into insulin producing cells [9].

Some protocols for differentiation of ES cells relied on the formation of embryoid bodies (EBs) to initiate differentiation. EB formation stimulates the chaotic differentiation of ES cells into all three germ lineages: endoderm, ectoderm and mesoderm. During EB formation *in vitro*, only a small fraction of differentiated definitive endoderm derived cells arise while a preponderance of ectoderm and mesodermal cells are generated. Since the large-scale production of uniform EBs represents a bioprocess engineering challenge and is not likely to yield pure populations of endoderm cells, strategies to overcome the need for EB formation are needed for establishing reliable and reproducible protocols for generating universal “off-the-shelf” tissues for transplantation.

During development, the visceral endoderm (primitive endoderm) and epiblast are derived from the inner cell mass of the blastocyst [10], [11]. In contrast to the visceral endoderm which is displaced to the extraembryonic sac, the epiblast cells ingress through the anterior segment of the primitive streak to form the definitive endoderm that eventually contributes cells that develop into the liver and pancreas [1], [10], [11], [12]. To distinguish definitive endoderm from other tissues, transcriptional factors such as Sry-related HMG-box transcription factor Sox17 [13], [14], the mix-like gene MIXL1 [13], [14], [15], and Foxa 2 (previously known as the hepatocyte nuclear factor (HNF) 3β) [16], [17], [18], [19] have been used as markers of definitive endoderm. An increase in expression of these definitive endoderm markers (Sox 17, GSC, Foxa 2 (HNF3β) and MIXL1) in combination with a concomitant decrease in expression of primitive endoderm marker (Sox 7), mesoderm markers (brachyury, MEOX1) and ectoderm markers (Sox 1 and ZIC1) have been collectively used as evidence for production of definitive endoderm.

Numerous factors have been proposed to promote definitive endoderm differentiation. For example, high concentrations of Activin A, a member of the transforming growth factor-beta (TGF-β) superfamily have been reported to promote endoderm formation [7], [13], [20], [21]. All-*trans*-retinoic acid (RA) has been shown to stimulate endoderm differentiation in teratocarcinoma stem cells and this effect was potentiated by addition of dibutyryl cyclic-AMP (DBcAMP) [22]. Retinoic acid has been implicated in embryonic endodermal patterning, especially during early pancreas formation [23], [24].

We hypothesized that by using a combination of all-*trans*-retinoic acid, basic fibroblast growth factor (bFGF) and dibutyryl-cyclic AMP, mouse embryonic stem cells can be differentiated into tissues that express characteristics of definitive endoderm that may serve as precursors for pancreatic endocrine cells.

## Materials and Methods

All tissue cultures with ES cells and experiments involving animals and their tissues were approved by the institutional animal care and research ethics committees.

### Generation of embryonic stem cells

ES cells were generated from C57BL/6 mouse 3.5 day post coitum blastocyst stage embryos by plating the embryos into a 96 well dish with irradiated feeder cells (primary embryonic fibroblasts, PEFs) and ES cell media consisting of high glucose containing Dulbecco's modified Eagle media (DMEM) supplemented with 15% fetal bovine serum (FBS), 100 U/ml+100 mg/ml penicillin (100 U/ml)-streptomycin (100 µg/ml), 100 µM 2-mercaptoethanol, 2 mM glutamine, 1 mM sodium pyruvate, 0.1 mM non-essential amino acids and 10 ng/ml Leukemia Inhibitory Factor [4] in the presence of 25 µg of PD98059 as described by Buehr and Smith [25]. Blastocysts were incubated at 37°C with 5% CO_2_. Approximately 2–3 days after plating, when the blastocysts have adhered and the cells have started to grow out, they were trypsinized and replated in ES cell media**.**

### Cell Culture and Differentiation

#### Step 1: Embryonic Stem Cell Maintenance

**Figure 1:**
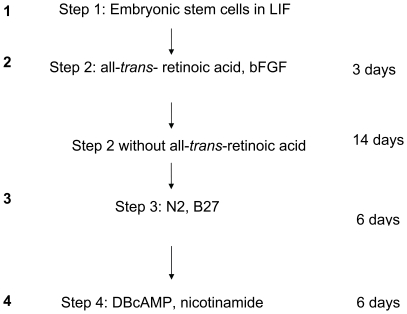
An overview of the culture protocol. The ES cells were taken out of LIF, and plated in the presence of all-trans-retinoic acid and bFGF (Step 2, denoted by 2) for 3 days followed by media without the retinoic acid but in the presence of bFGF for 14 days. These cells were then grown in suspension in the presence of N2 and B27 for 6 days (Step 3, denoted by 3) before being put into media containing DBcAMP and nicotinamide for 6 days (Step 4, denoted by 4).

C57BL/6 mouse ES cells were cultured on a feeder layer of gamma irradiated PEFs and incubated at 37°C with 95% O_2_/5% CO_2_ in an ES cell maintenance medium comprised of 15% FBS (Gemini Bio-Products, Woodland, CA), 1 mM sodium pyruvate (Stem Cell Technologies, Vancouver, Canada), 2 mM glutamine (Stem Cell Technologies), 0.1 mM non-essential amino acids (Stem Cell Technologies), 10 ng/ml leukemia inhibitory factor (Chemicon International, Temecula, CA), 100 µM 1-thioglyercol (MTG) (Sigma, St. Louis, MO) and high glucose DMEM (Stem Cell Technologies) for 2–3 days until they were 70–80% confluent. The cells from this step are represented with “1” in Figure 1 and throughout other figures.

#### Step 2

As illustrated in Figure 1, ES cells were dissociated with trypsin/EDTA solution and plated onto 100 mm gelatinized tissue culture dishes in 1∶10 dilutions and incubated at 37°C with 95% O_2_/5% CO_2_ with 15% FBS, 0.1 mM non-essential amino acids, 1 mM MTG, 2 mM of L-glutamine, 10^−7^ M of all-*trans*-retinoic acid (Sigma) and 25 ng/ml of human basic fibroblast growth factor (bFGF) (Stem Cell Technologies), and high glucose DMEM for 3 days. The cells are represented by the number 2.

#### Step 3

The cells generated in step 2 were then maintained at 37°C with 95% O_2_/5% CO_2_ with 15% FBS containing high glucose DMEM supplemented with 0.1 mM non-essential amino acids, 1 mM MTG, 2 mM of L-glutamine in the presence of 25 ng/ml of bFGF for up to 14 days. At this time the cells were trypsinized, harvested, plated onto petri dishes, and incubated for 6 days in suspension at 37°C with 95% O_2_/5% CO_2_ with ES-Cult basal medium-A with 1x N2 supplement-A (Stem Cell Technologies), bFGF, 1x B27 supplement (Stem Cell Technologies) to facilitate formation of cell clusters (represented by number 3).

#### Step 4

The cell clusters from Step 3 were cultured for 6 days at 37°C with 95% O_2_/5% CO_2_ in the presence of 2×10^−3^ M dibutyryl cAMP in ES-Cult basal medium-A supplemented with 1x N2 supplement and 10 mM nicotinamide (represented by number 4).

### Immunohistochemistry

The Step 4 cell clusters were fixed in 4% formalin and subsequently embedded in paraffin. The paraffin blocks were deparaffinized in xylene, rehydrated in graded ethanol and washed in phosphate buffered saline (PBS). Antigen retrieval was necessary prior to immunostaining for most antibodies (10 mM citrate buffer; pH 6.0). Sections were incubated for 1 hour with blocking buffer containing 2–5% serum followed by incubation with primary antibodies for 1 hour at room temperature or overnight at 4°C. Sections were washed and incubated with respective secondary antibodies for 1 hour at room temperature. The primary antibodies used were rabbit anti-mouse PDX-1 (1∶500, Chemicon, Temecula, CA), rabbit anti-mouse Ngn3 (1∶500, a gift from Dr. F. Lynn), mouse anti-carboxypeptidase E (CPE) (1∶500, BD Biosciences, San Jose, CA), rabbit anti-pan-cytokeratin (1∶100, Santa Cruz Biotechnology, Santa Cruz, CA), guinea pig anti-human insulin (1∶100, Dako, Glostrup, Denmark), rabbit anti-human glucagon (1∶75, Dako), goat anti-rat antibody for C-peptide made with purified synthetic rat C-peptide II (1∶100, Linco Research, St. Charles, MO), mouse anti-mouse somatostatin (1∶500, a gift from Dr. C. McIntosh, Vancouver, Canada), guinea pig anti-rat pancreatic polypeptide (1∶1000, Linco), goat anti-human ghrelin (1∶100, Santa Cruz) and rabbit anti-human amylase (1∶2000, Sigma). The secondary antibodies used included Alexa 594 or 488 anti-guinea pig, mouse, rabbit, or rat (1∶200, Jackson Immuno Research, West Grove, PA). All antibodies were diluted in PBS with 1% BSA or in blocking buffer (PBS with 2–5% serum).

### Quantitative PCR (Q-PCR)

RNA was isolated from cells in each step using TRIZOL reagent (Invitrogen, Burlington, ON, Canada) according to the manufacturer's instructions. The isolated RNA was treated with DNAse (Invitrogen) and quantified with an Ultrospec® 3000 spectrophotometer (Pharmacia Biotech Ltd, Cambridge, UK). The primers used are listed in Table 1. In order to demonstrate enrichment for definitive endoderm relative to other germ lineages the following transcripts were analyzed: Sox 17 and Foxa 2 for definitive endoderm; brachyury for mesoderm; Sox 7 for primitive endoderm; and Sox 1 for ectoderm. Marker of pluripotency, Oct-4 was used to document the extent of differentiation. The markers of pancreatic precursors and differentiating beta cells, Nkx6.1, PDX-1 and Insulin I and II, were used to look for evidence of pancreatic lineage. To further verify beta cell lineage differentiation, levels of islet amyloid polypeptide (IAPP) and islet-specific glucose-6-phosphatase (IGRP) were also measured. PAX 6 was used as a pre-beta cell endocrine cell marker [26], [27]. Amylase was used as a marker of exocrine pancreas. PAX 6 and neurogenin (Ngn) 3 were used as endocrine markers. Prevalidated primers for Sox 7, Sox1, brachyury, Ngn3 and PDX-1 were purchased from Applied Biosystems (Foster City, CA). All other primers were designed using Primer3 (http://frodo.wi.mit.edu/cgi-bin/primer3/primer3_www.cgi) and spanned introns where possible (Table 1). Amplicons were between 80–120 bp for efficient amplification. Primer efficiencies were determined using an exponential cDNA dilution series. Only primer pairs with an efficiency greater than 0.8 were used in subsequent analyses. A 7500 Real Time PCR System (Applied Biosystems) and SYBR Green PCR master mix (Applied Biosystems) were used for all reactions. cDNAs were generated by reverse transcription (RT) of 1 µg of total RNA from newly isolated tissue for each RT. 10 ng of generated cDNA was used in each reaction with all reactions done in duplicate. Samples were normalized to beta-actin, and the fold increase compared to Step 1 as calculated using 2^−ΔΔCT^ [28] as appropriate unless otherwise noted.

**Table 1:** A list of primers for the various markers.

L-Amylase	CCAAGGAAGCAGACCTTTCA
R-Amylase	ACACGGCCATTTCCAAAGTA
L-β-actin	GCTCTTTTCCAGCCTTCCTT
R-β-actin	CGGATGTCAACGTCACACTT
L-Foxa2	CATCCGACTGGAGCAGCTA
R-Foxa2	TGTGTTCATGCCATTCATCC
L-IAPP	CCTCCTCATCCTCTCTGTGG
R-IAPP	GCACTTCCGTTTGTCCATCT
L-IGRP	AGGGGACTGGTTCAATCTCA
R-IGRP	TGGGCTTGAATGATTTGGAT
L-Insulin I	CTTCAGACCTTGGCGTTGGA
R-Insulin I	ATGCTGGTGCAGCACTGATC
L-insulin II	GGCTTCTTCTACACACCCATGT
R-insulin II	GGTCTGAAGGTCACCTGCTC
L-Nkx6.1	CCCGGAGTGATGCAGAGT
R-Nkx6.1	TTCTCTTCCCATGTTTGTCCA
L-Oct4	GTTGGAGAAGGTGGAACCAA
R-Oct4	TCTTCTGCTTCAGCAGCTTG
L-PAX6	TCAGCAGCAGCTTCAGTACC
R-PAX6	CCCAACATGGAACCTGATGT
L-Sox17	CTTTATGGTGTGGGCCAAAG
R-Sox17	GGTCAACGCCTTCCAAGACT

### Insulin Release Assay

The Step 4 cell clusters, recovered in insulin free, low glucose recovery medium composed of Ham's F12/DMEM (no glucose) (Gibco/Invitrogen) and 10 mM nicotinamide overnight, were washed in Krebs-Ringer bicarbonate buffer (KRBB) with 0.1% BSA in 1.67 mM D-glucose. Total insulin content of the cells was determined by lysing of cells in 1 M glacial acetic acid and quantifying insulin levels by radioimmunoassay. In a separate experiment, to assess for glucose responsive insulin release from the cluster, the clusters were first washed in KRBB with 0.1% BSA in 1.67 mM D-glucose and then exposed to either 1.67 mM or 16.7 mM D-glucose in KRBB with 0.1% BSA for 2 hours. Insulin radioimmunoassay (RIA) was performed to determine the levels of murine insulin I and II using a human insulin radioimmunoassay kit according to the manufacturer's instructions (MP Biomedicals, Irvine, CA). The insulin content was calibrated per µg DNA (determined using an Ultrospec® 3000 spectrophotometer; Pharmacia Biotech Ltd, Cambridge, UK).

### Transmission Electron Microscopy

To assess whether these cells have mechanisms for secretion, transmission electron microscopy [29] was performed to look for secretory granules in the cytoplasm. The step 4 clusters were fixed in EM grade 4% formaldehyde and embedded in Lowicryl K4M resin. Ultra thin sections were prepared on an RMC MT-6000-XL ultramicrotome (Sorvall, Microtomes, Wilmington, DE) and examined for the presence of secretory granules with Hitachi H7600 transmission electron microscope.

## Results

### Endoderm Differentiation

Production of definitive endoderm from murine ES cells was assessed in three different ways. First, the markers of definitive endoderm (Sox 17 and Foxa 2) were quantified in the different steps of the protocol. Second, the marker of primitive endoderm, Sox 7, was measured to ensure that any observed expression of Sox 17 and Foxa 2 did not arise from primitive endoderm where these two markers may also be expressed. Finally, levels of Sox 1 (neuroectoderm marker) and brachyury (mesoderm marker) were measured to determine if there was preferential differentiation to the definitive endoderm lineage.

**Figure 2:**
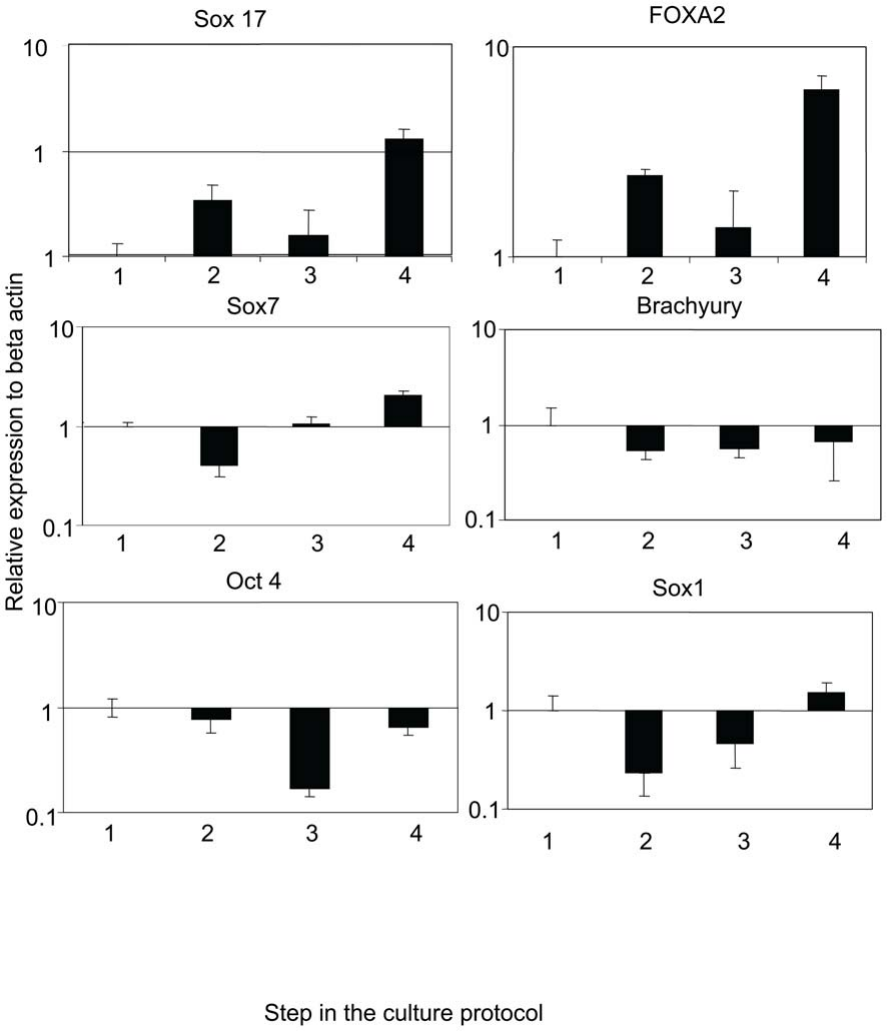
Quantitative PCR results of definitive endoderm markers (Sox 17 and FOXA2), mesoderm marker (brachyury), neuroectoderm marker (Sox 1), primitive endoderm marker (Sox 7), and pluripotency marker (Oct 4) in the cells cultured by the protocol. The expression of each gene is illustrated as expression of SYBR green (semi-log scale on Y axis) relative to its level in ES cells (1).

As illustrated in Figure 2, there was an upregulation of definitive endoderm markers: Sox17 (13 fold) and Foxa 2 (6 fold) in the latter steps of the protocol relative to their respective expression in embryonic stem cells (Step 1). In contrast, the primitive endoderm marker Sox 7, showed only a marginal increase in expression in steps 3 and 4. Moreover, the Sox 7 primer consistently produced high Ct values (>30) suggesting that only nominal levels of this gene were expressed. Similarly, the mesoderm marker brachyury and the neuroenctoderm marker Sox 1 did not increase appreciably and produced high Ct values. The marker of pluripotent cells, Oct-4 was expressed at very low levels throughout the protocol (Figure 2) supporting decreased pluripotency of the cells and adequate differentiation away from the embryonic stem cell stage.

Taken together, the quantitative PCR results suggest that the protocol here produces definitive endodermal tissues from murine ES cells. These observations are consistent with those of D'Amour et al. [13] and suggest that the production of definitive endoderm using this approach arises from a more preferential differentiation rather than a differentiation as a part of all three germ layers. It is possible that some of the expression of the definitive endoderm markers have been contributed by extraembryonic endoderm. There is evidence in the literature that the combination of RA and DBcAMP can induce production of extraembryonic endoderm [30], [31], [32], [33]. However, based on the data, the contribution of Sox 17 and Foxa 2 by primitive endoderm is likely minimal.

### Production of Cells of Pancreatic Lineage

#### Quantitative PCR and Immunohistochemistry

The generation of cells of the pancreatic lineage was assessed by measuring expression levels of the following markers: IAPP, IGRP, insulin I and insulin II (beta cell markers), the transcriptional regulators Nkx 6.1 and PDX-1 (pancreas development markers) and an endocrine marker, PAX 6 using Q-PCR. Another pancreatic endocrine marker Ngn3 was expressed at increasing levels throughout the differentiation protocol. In addition, fluorescence immunohistochemistry was performed to determine cellular protein levels of insulin, glucagon, C-peptide, PDX-1, Ngn-3, carboxypeptidase E (CPE), pan-cytokeratin (pan-CK), somatostatin, pancreatic polypeptide and ghrelin.

**Figure 3:**
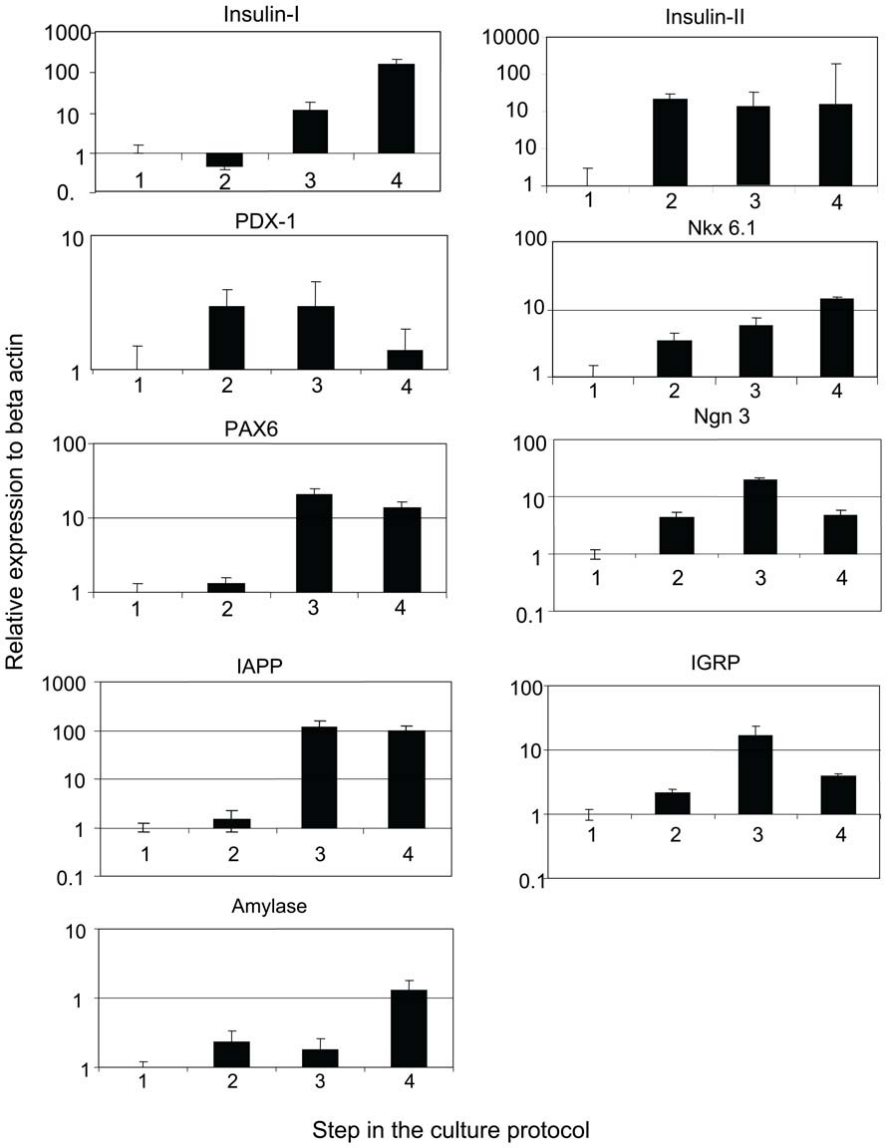
Quantitative PCR results of pancreatic markers (insulin-I, insulin-II, IAPP, IGRP, amylase, PDX-1, Nkx 6.1) and endocrine markers (PAX6 and Ngn 3) in the cells cultured by the protocol. The expression of each gene is illustrated as expression of SYBR green (semi-log scale in the Y-axis) relative to its levels in ES cells (1).

**Figure 4:**
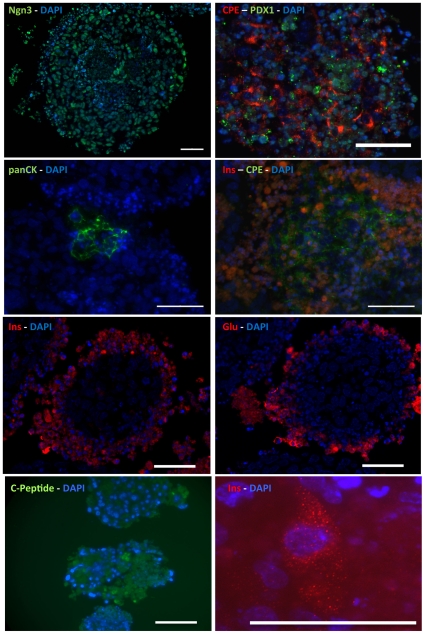
Immunohistochemistry of cell clusters for pancreatic endocrine markers insulin (Ins), C-peptide (CP), carboxypeptidase E (CPE), neurogenin 3 (Ngn3), PDX-1, and glucagon (Glu). Exocrine pancreatic marker pan-cytokeratin (panCK) was also present. The nuclear marker DAPI is included in each figure. Scale bar represents 50 um.

The Q-PCR analyses revealed an approximately 100 fold increase in expression of insulin I and insulin II in the last step of the protocol (Figure 3). Of note is the observation that insulin I, which is produced only in the pancreas, progressively increased with each step. In contrast, insulin II, which is produced not only in the pancreas, but also in fetal liver, neurons and yolk sac [34], [35], was expressed at a constant level throughout steps 2–4. In keeping with the Q-PCR results, a small proportion of the cells in the step 4 clusters were positive for insulin (Figure 4). Both Nkx6.1 and PAX 6, markers of endocrine differentiation increased throughout the differentiation process. Other beta cell markers IAPP and IGRP showed an increase with the differentiation steps. There was some production of exocrine pancreatic tissue as evidenced by an increased production of amylase in the last step of the protocol (Figure 3). In contrast, PDX-1 levels were increased during steps 2 and 3, prior to decreasing during step 4.

As illustrated in Figure 4, many of the cells in the core of the cell cluster were necrotic, likely from the fact that these were cultured in suspension and the delivery of nutrients in the media was compromised to the cells in the inner core. Most clusters express Ngn3, whereas the expression of PDX-1 was markedly lower. A small proportion of cells were positive for islet markers such as insulin, C-peptide and glucagon. Also, a small proportion of the cells expressed CPE and pan-CK. CPE was used as an additional islet marker and pan-CK was used as an exocrine pancreatic marker (ductal). Notably, the CPE staining was cytoplasmic and punctate, compatible with granular localization, whereas PDX-1 stain was perinuclear in distribution as expected. There was a progressive increase in the expression of amylase and this implies production of exocrine pancreatic tissues. The cell clusters did not express somatostatin, pancreatic polypeptide or ghrelin (data not shown).

#### Characterization of the step 4 cells

**Figure 5:**
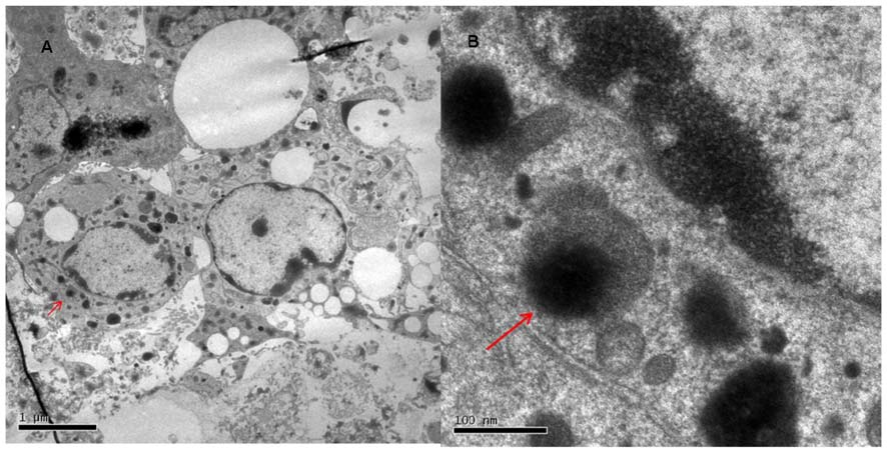
Transmission electron microscopy of the Step 4 clusters in (a) low power and (b) high power magnification. There are secretory granules present in the cytoplasm of the cells denoted by arrows.

To characterize the step 4 cells, insulin (insulin I and II) content was quantified. The insulin radioimmunoassay used reliably detects both insulin I and II. The Step 4 cell clusters had a total insulin content of 50.4±7.1 µIU/ml/µg of DNA. As outlined in Table 2, the stimulated insulin release assay revealed high basal levels of insulin secretion (17.2±3.1%) at low glucose concentrations (1.67 mM) with a significant increase at high glucose concentrations (16.7 mM), (29.3±1.1%, p<0.03). To assess the step 4 clusters for cells that possess mechanisms for secretion, TEM was performed on these cells to identify secretory granules in the cytoplasm. As illustrated in Figure 5, these cells have mulitple secretory granules.

**Table 2:** Glucose-stimulated insulin release.

Cell Clusters	% Insulin Secretion Glucose Concentrations		P value
	1.67 mM	16.7 mM	
Step 4 (4)	17.2±3.1	29.3±1.1	0.03

Total insulin (insulin I and II) secretion in response to exposure to low (1.67 mM) and high (16.7 mM) glucose concentration for the cell clusters from Step 4 (4). Student's t-test was used to determine the differences in percent secretion.

## Discussion

Generation of definitive endoderm from ES cells is the first essential step toward generation of pancreatic tissues. In this manuscript, we describe a protocol that has produced a population of cells that express characteristics of definitive endoderm. This protocol does not require formation of EBs, but culturing of cells in a monolayer. The potential advantages of the monolayer may include uniform exposure of cells to the reagents in the media and enhanced ease of handling of the cells. This may possibly help reproducibility and facilitate process scale up.

Since the work by D'Amour et al. suggested a criteria for accepted markers of definitive endoderm [13], a combination of the increase of markers such as Sox 17, GSC, Foxa 2 and MIXL1 and a decrease in the primitive endoderm markers that are not expressed in definitive endoderm such as Sox 7 needs to be included to support the argument for production of definitive endoderm. Furthermore, a decrease in the markers expressed in other germ layers (i.e. ectoderm and mesoderm) would suggest that a particular protocol is able to enrich the cell population with definitive endodermal cells.

In this study, RA and DBcAMP appeared to induce production of definitive endoderm from ES cells when they were cultured with serum and bFGF for an extended period of time as evidenced by an expression of Sox 17 and Foxa 2. Also, there were minimal levels of Sox 1 and brachyury suggesting that there was no significant production of neuroectodermal and mesodermal tissues. This observation is in keeping with previous studies indicating that RA plays an important role in the development of the murine dorsal pancreas [36] and induces production of PDX-1 positive endoderm [37] in an ES cell differentiation model that involves EB formation. Further evidence that RA facilitates this differentiation process is the observation that Fibroblast growth factor 2 (FGF2) binds transmembrane fibroblast growth factor receptors and plays a key role in mouse embryogenesis, including development of the pancreas and liver [38], [39], [40]. FGF2 signaling has also been suggested to play an important role in development of the exocrine pancreas [41] and in maintenance of adult beta cell function [42]. In liver development, FGF2 signaling from the cardiac mesoderm induces hepatogenesis [43], and furthermore, FGF2 signaling appears to stimulate expression of sonic hedgehog which in turn inhibits pancreatic development [38]. FGF2 has been shown to be important in pancreatic development and maintenance of beta cell function, suggesting that FGF2 may promote pancreatic differentiation. In a separate experiment, exposing the cells to FGF2 for a shorter period of time resulted in lower insulin content in cells (data not shown).

During normal development, mature beta cells develop after formation of definitive endoderm, and the glucose responsive insulin release is a feature of mature beta cells. The expression of definitive endoderm markers increased throughout the four steps of the protocol. In addition, the expression levels of several markers indicative of pancreas differentiation, including Nkx6.1, Pax6 and insulin I, also progressively increased whereas the expression of Pdx-1, a transcriptional regulator that plays critical roles in pancreas specification and development, as well as beta cell function appeared to peak at step 3 and then decreased during step 4. Collectively, these data suggest that the differentiation protocol promoted the progressive maturation of ES cells towards definitive endoderm and pancreatic lineage. The expression of Nkx6.1, PAX 6, Ngn3, IAPP, IGRP, CPE, Insulin I, C-peptide and PDX-1 in the cell population suggests that a subpopulation of cells in Step 4 were endocrine cell precursors. Furthermore, Step 4 cells also contained a small subset of mature glucose responsive insulin producing cells as supported by the immunohistochemistry and insulin release data.

In summary, the ES cell differentiation protocol described in this study resulted in the generation of cells that displayed characteristics of definitive endoderm. There is a production of a small number of heterogeneous population of cells that express characteristics of pancreatic tissues; however, most cells appear to be in the pancreatic endocrine precursor stage. The insulin production from the cells in this protocol is not significant and further analysis is required to elucidate the nature and the origin of the insulin producing cells. Studies are underway to assess the efficiency of the protocol for endoderm production.
